# Influence of Fermentation Temperature and *Metschnikowia pulcherrima/Saccharomyces cerevisiae* Multi-Starter Cultures on the Volatile Compounds of Lugana Wine

**DOI:** 10.3390/foods14203538

**Published:** 2025-10-17

**Authors:** Giulia Bertazzoli, Emma Pelizza, Giovanni Luzzini, Giovanna E. Felis, Maurizio Ugliano, Sandra Torriani

**Affiliations:** 1 Department of Biotechnology, University of Verona, 37134 Verona, Italy; giulia.bertazzoli@univr.it (G.B.); emma.pelizza@univr.it (E.P.); giovanni.luzzini@univr.it (G.L.); giovanna.felis@univr.it (G.E.F.); maurizio.ugliano@univr.it (M.U.); 2 VUCC-DBT, Verona University Culture Collection, University of Verona, 37134 Verona, Italy

**Keywords:** multi-starter cultures, *Metschnikowia pulcherrima*, fermentation temperature, white wine, volatile profile, energy savings, sustainability

## Abstract

Low fermentation temperatures are a crucial aspect of winemaking, influencing yeast metabolism, process efficiency, and aroma retention. However, it is a significant source of energy consumption. Here, we investigated the effect of high (20 °C ± 1 °C) and low (16 °C ± 1 °C) temperatures combined with multi-starter fermentations with *Metschnikowia pulcherrima* (Level^2^ Flavia and Level^2^ Initia) and *Saccharomyces cerevisiae* (EC 1118 and Zymaflore X5) strains on the aromatic profile of Lugana wine. Sequential inoculation at 20 °C shortened fermentation by five days compared to 16 °C, lowering energy consumption for cooling. Significant differences in key aromatic compound classes were observed between the two temperature trials. Wines fermented at 20 °C exhibited more intense tropical and fruity aromas, with 3-MH and 3-MHA concentrations up to ten times higher than those at 16 °C. Such an increase was particularly evident in the sequential fermentation, especially Level^2^ Flavia/Zymaflore X5, compared to *S. cerevisiae* alone. In contrast, lower temperatures favored the production of ethyl esters and ethyl acetate, with increases of 14% for ethyl octanoate and 24% for ethyl butanoate. Sensory analysis confirmed these aromatic differences, with all trials at 20 °C enhancing white wine aroma complexity, particularly those involving multi-starter fermentations. These findings provide practical insights for the wine industry, validating sequential fermentation at moderately elevated temperatures as a strategy to improve wine quality while supporting energy-efficient and sustainable practices.

## 1. Introduction

In recent years, environmental sustainability has become a key issue for the wine industry due to increasing awareness of the environmental impacts of intensive resource utilization and greenhouse gas emissions associated with agricultural practices [1]. The wine production chain, from grape harvest to bottling, is estimated to contribute approximately 0.3% of annual greenhouse gas emissions from human activities worldwide [2]. To meet the growing demand for sustainable production, winemakers have adopted various practices to reduce their environmental footprint while maintaining production efficiency [2]. Among these strategies, the use of specific selected yeasts, capable of fermenting with desirable sensory properties at higher temperatures than standard ones, offers potential enhancement of both product quality, chemical input reduction and fermentation optimization, as well as reduction of cooling during fermentation [3,4]. Indeed, cooling systems and temperature control during the fermentation process represent a significant source of energy consumption, highlighting the urgent need to improve energy efficiency [5,6].

It is well-known that temperature plays a crucial role in fermentation processes, influencing yeast metabolism and retention of aroma in the final product [7,8,9,10]. At lower fermentation temperatures (around 15 °C), yeasts produce higher concentrations of compounds associated with fresh and fruity aromas. Therefore, cooler environments are traditionally chosen to preserve these volatile compounds and reduce their evaporative loss, especially in young white wines [8,11]. On the other hand, higher fermentation temperatures (28 °C) are more conducive to producing flowery aroma compounds [10]. This production is due to the differential expression of genes involved in aroma compound metabolism at various temperatures [10]. In addition, temperature impacts the yeast growth rate and the overall fermentation kinetics. Higher temperatures generally accelerate fermentation but can also lead to the loss of some volatile compounds. Consequently, proper temperature setting and control during fermentation are crucial to achieving the desired sensorial quality of wine and improving environmental sustainability by minimizing energy consumption [6].

The influence of temperature has been explored mainly on the fermentation kinetics and aroma release of *Saccharomyces cerevisiae* [9,12,13]. At the same time, fewer studies on this aspect have been conducted on *S. cerevisiae*/non-*Saccharomyces* yeast mixed fermentations. The use of non-*Saccharomyces* yeasts as co-starters in winemaking received increasing attention for their potential to produce wines with greater complexity and adaptability to changing environmental conditions [14,15,16]. They were reported to possess aroma-related enzymatic activities and other metabolic traits that are not present or are less pronounced in *S. cerevisiae* (for example, β-glucosidase activity); thus, they can be considered positive resources for facing the challenges of evolving consumer demands and environmental issues [16,17,18]. However, non-*Saccharomyces* yeasts are generally unable to complete alcoholic fermentation due to their poor ethanol tolerance; for this reason, they are usually used in mixed inoculum with *S. cerevisiae* [4,6,7].

Among the non-*Saccharomyces* yeast species with high potential to release aroma compounds, *Metschnikowia pulcherrima* is one of the best studied [19,20]. Indeed, this yeast exhibits distinctive genetic and phenotypic characteristics, including intense extracellular enzymatic activity [16]. Particularly, its β-glucosidase and β-lyase activities can exploit the varietal aromas of wine, typically defined as terpenes and thiols [13,21]. These volatile compounds are derived from their odorless, non-volatile glycoside precursors, released during wine fermentation by different yeast hydrolytic enzymes [20].

As for terpenes, diverse enzymes act sequentially to hydrolyze disaccharide glycosides according to two steps: firstly, a specific exo-glycosidase, such as α-l-arabinofuranosidase, β-d-apiofuranosidase, and α-l-rhamnopyranosidase, breaks the bond between the respective sugar moiety and the β-d-glucoside, releasing one molecule of arabinose, apiose, or rhamnose, respectively; in the 2nd step, the β-glucosidase action on the remaining glucoside releases the free aromatic aglycone and glucose. Although β-glucosidase is considered a typical feature of *M. pulcherrima* [16,22,23,24], this characteristic is highly strain-dependent and can be influenced by the substrate used for testing [16,25]. A recent survey on an extensive collection of *M. pulcherrima* strains isolated from diverse sources [26] evidenced that only 41.5% of the 193 strains tested expressed high β-glucosidase activity on a medium containing arbutin. Therefore, a proper selection of positive-β-glucosidase *M. pulcherrima* starters is crucial for improving the varietal aroma of wine.

Varietal thiols, such as 4-mercapto-4-methylpentan-2-one (4MMP), 3-mercaptohexan-1-ol (3MH) and its acetate form, are key aroma compounds produced by yeast metabolism that impart tropical and citrus notes to wine. These thiols are released during fermentation from odorless cysteine S-conjugate precursors through enzymatic reactions involving β-lyases [16,27]. This process is highly inefficient, with less than 5% of precursors converted to thiols [28,29]. For example, the enzyme Str3p releases only about 0.1% of free 3MH and 0.6% of 4MMP compared to its activity against l-cystathionine [30]. It is documented that some *M. pulcherrima* strains present β-lyase activity [23]. This highlights the challenge of increasing thiol release, a process significantly influenced by fermentation parameters, such as temperature, and emphasizes the potential opportunity to enhance aromatic expression in wine through strain selection [2,21].

In this context, here we investigated the impact of the fermentation temperature (16 °C ± 1 °C and 20 °C ± 1 °C) combined with the use of commercial *M. pulcherrima* and *S. cerevisiae* strains in sequential inoculations and *S. cerevisiae* strains in pure culture on the aromatic quality of Lugana wine.

## 2. Materials and Methods

### 2.1. Yeast Strains

*Metschnikowia pulcherrima* Level^2^ Flavia and Level^2^ Initia (hereinafter named M1 and M2, respectively) were used to conduct microvinification trials as co-starters with *Saccharomyces cerevisiae* EC 1118 or Zymaflore X5 (hereinafter named S1 and S2, respectively). The producers and some distinctive enological features of these yeasts are reported in Table 1.

The active dry yeasts were rehydrated in sterile distilled water following the manufacturer’s instructions, and viable cell counts were evaluated on WL (Wallerstein Laboratory) Nutrient Agar (Oxoid, Freiburg, Germany) plates incubated at 27 °C for 48 h. After counting, colonies from each preparation were isolated and cultured in YPD broth (10 g/L yeast extract, 20 g/L peptone, 20 g/L glucose, 20 g/L agar). The pure cultures were maintained at −80 °C under cryo-preservation in the Department of Biotechnology Culture Collection.

### 2.2. Enzymatic Assays

Enzymatic activities were evaluated by spot assay on Petri dishes filled with appropriate differential media. A droplet of a 24-h YPD culture was deposited onto the agar surface and dried under a biosafety cabinet. The yeast strains tested were inoculated in quadruplicate. Plates were incubated at 27 °C for 72 h.

The β-glucosidase activity was determined as reported by [34] on the esculin agar medium containing 2 g/L glucose, 1 g/L peptone, 1 g/L yeast extract, 3 g/L esculin, 0.01 g/L ferric ammonium citrate, and 15 g/L agar; the pH was adjusted to 5.0, and the medium was sterilized by autoclaving. Following sterilization, an additional 20 mL/L of filtered 1% (*w*/*v*) ferric ammonium citrate solution was added. The presence of the enzymatic activity was visualized as a dark halo around the yeast spot growth. It is reported that the commercial *M. pulcherrima* strain Level^2^ Flavia expresses α-arabinofuranosidase and β-glucosidase, releasing volatile terpenes that enhance fruity aromas in certain grape varieties [22]. The β-lyase activity was assessed using the YCB-SMC medium developed by [23], having the following composition: 0.1% S-methyl-L-cysteine, 0.01% pyridoxal-5-phosphate, 1.2% Yeast Carbon Base, and 2% agar, pH adjusted to 3.5. The growth of the colonies after incubation indicated the presence of β-lyase activity. The strains were serially cultivated twice in the YCB-SMC medium to avoid false positives.

### 2.3. Microvinification Trials

Microvinifications were carried out with must from Trebbiano grapes harvested during vintage 2024 at Cantina Delibori, Lonato del Garda (BS), Italy. Trebbiano is a white *Vitis vinifera* cultivar mainly grown south of Lake Garda (Verona, northern Italy), typically fermented at low temperatures (16 °C) to produce Lugana wine. Its grapes contain glycosidic, cysteinylated, and glutathionylated aroma precursors, which serve as the backbone for the tropical and fruity notes characteristic of Lugana wine. The grapes were destemmed and crushed, and the must was kept at 4 °C for 24 h for must clarification. The must had received no thermal treatment or addition of SO_2_ and was kept refrigerated until yeast inoculation.

Sterile 500 mL glass bottles were filled with 300 mL of must and closed with perforated silicon stoppers and two pipette tips to allow the carbon dioxide to release and prevent contamination.

Fermentation trials were conducted at two temperatures, 16 °C ± 1 °C and 20 °C ± 1 °C, by inoculating six different starter treatments, including:

(1) control *S. cerevisiae* EC 1118 pure culture (S1), inoculated at time 0;
(2) control *S. cerevisiae* Zymaflore X5 pure culture (S2), inoculated at time 0;
(3) inoculation of *M. pulcherrima* Flavia followed by *S. cerevisiae* EC 1118 after 48 h (M1S1);
(4) inoculation of *M. pulcherrima* Flavia followed by *S. cerevisiae* Zymaflore X5 after 48 h (M1S2);
(5) inoculation of *M. pulcherrima* Initia followed by *S. cerevisiae* EC 1118 after 48 h (M2S1);
(6) inoculation of *M. pulcherrima* Initia followed by *S. cerevisiae* Zymaflore X5 after 48 h (M2S2).

Each yeast strain was inoculated at approximately 1 × 10^6^ UFC/mL. Thus, six different trials were conducted, in triplicate, at two temperatures, for a total of 36 fermentations, considering 24 sequential fermentations of *M. pulcherrima*/*S. cerevisiae* and 12 single-strain fermentations with *S. cerevisiae* strains to be used as controls.

### 2.4. Fermentation Kinetics and Microbiological Analysis

Fermentation kinetics were monitored daily by measuring bottle weight loss due to CO_2_ release. Each fermentation was stopped when daily weight loss dropped below 0.05 g/L by adding 100 mg/L of potassium metabisulfite (K_2_S_2_O_5_). After alcoholic fermentation, wine was centrifuged (9500× *g*, 20 min) at 4 °C and bottled without headspace by flushing nitrogen to prevent oxidative phenomena with crown caps. The bottles were then stored in a cold room at 16 °C until aromatic analyses were performed.

Through fermentation, cell concentration was determined using plate counts on WL nutrient agar, and the results were expressed as the mean of two values. After 48 h of incubation at 27 °C, yeast cells were counted differently based on the morphological differences distinguishing non-*Saccharomyces* yeast species from *S. cerevisiae* [35].

To confirm the implantation of the starters, colonies of putative *M. pulcherrima* and *S. cerevisiae* were randomly isolated from each WL Nutrient Agar plate and grown in YPD broth at 27 °C for 48 h. Two millilitres from each culture were centrifuged (14,000× *g*, 5 min), and the cell pellet was used for DNA extraction. The total genomic DNA of isolates was purified using the Wizard Genomic DNA Purification kit (Promega, Milano, Italy), following the manufacturer’s protocol. The dominance of the *M. pulcherrima* starters was assessed using the fingerprinting method with the combination of (GTG)_5_ and M13 primers, following the PCR protocol of [26]. The dominance of *S. cerevisiae* starters was assessed using the Interdelta fingerprinting method, which exploits the delta elements that form the flanking retrotransposons of LTR TY1 and TY2, through the PCR protocol of [36]. PCR products were run on 2% (*w*/*v*) agarose gel in 1× TAE buffer, stained with EuroSafe colorant Acid Stain at 110 V. Visualization and image capturing were made under UV with a ChemiDocXRS+ Imaging System (Bio-Rad, Segrate, Italy).

### 2.5. Chemical Analysis of Must and Wine

Must and wine samples were centrifuged (5000× *g*, 10 min), and the clarified supernatants were analyzed using the automated Analyzer Y15 (BioSystems S.A., Barcelona, Spain). Specific enzymatic kits were used to measure the following parameters in grape must: sugars (glucose and fructose), primary amino nitrogen (PAN), ammonium, and total acidity. Yeast assimilable nitrogen (YAN) was calculated as the sum of PAN and 0.8225 × ammonium. Besides these parameters, acetic acid, acetaldehyde, glycerol, free sulfites, and total sulfites were measured in wine samples. Ethanol was analyzed with an Alcolyzer FTIR Lyza 5000 (Anton Paar, Graz, Austria).

### 2.6. Quantification of Volatile Compounds

Volatile Organic Compounds (VOCs) were quantified by gas chromatography coupled to mass spectrometry (GC–MS) following the procedure described by [37]. The extraction of VOCs from wine samples was carried out using solid phase microextraction (SPME). The GC-MS analysis was performed on an HP 7890A gas chromatograph (Agilent Technologies, Santa Clara, CA, USA) coupled to a 5977B quadrupole mass spectrometer equipped with an MPS3 autosampler (Gestel, Müllheim an der Ruhr, Germany). Separation was performed following the protocol described by [21].

For the quantification of the volatile thiols 3-mercaptohexanol (3-MH) and 3-mercaptohexylacetate (3-MHA), the extraction method for LC-MS/MS developed by [38] was used. The protocol is as follows: 40 mL of the wine sample was mixed with 150 µL of internal standard solution (4-methoxy-α-toluenethiol, 350 mL/L in ethanol) in a 50 mL Falcon tube. Dichloromethane (6 mL) was added, and the mixture was shaken for 1 min. The sample was centrifuged at 9500 rpm for 30 min, and the dichloromethane phase was transferred from the Falcon tube to a glass test tube. 2 mL of the organic phase was transferred to a 4 mL amber vial and spiked with 150 µL of Ebselen ethanol solution (800 mg/L in ethanol). The obtained mixture was stirred, dried under nitrogen, and dissolved in 1 mL of methanol. The sample was then stored at −20 °C in a freezer. Before injection, the sample was filtered using a syringe and a 0.22 µm filter and then transferred to a vial with an HPLC cap. The same filter was used for replicating samples. LC-MS/MS separation was performed using the conditions of [38].

### 2.7. Sensory Evaluation

Sensory evaluation of the experimental wines was conducted using the sorting task methodology, adapted from the approach outlined by [39]. The sessions included twelve judges (five men and seven women) who were wine science researchers or teaching staff actively involved in winemaking and wine evaluation. All participants were classified as wine experts according to the criteria set by [40]. Before the test, samples were taken out of the 16 °C cold room one hour in advance, and 20 mL of each wine was poured into ISO wine glasses [41]. The glasses were labelled with 3-digit random codes and covered with plastic Petri dishes. All samples were served at room temperature, and the order of the glasses was randomized for each panelist. Participants were instructed to sort the wines into groups based on olfactory similarities using orthonasal evaluation only, without specifying odor descriptors. They were allowed to create as many groups as needed. A trained panel performed sensory analyses; by local regulations, ethical committee approval was not required for this study.

### 2.8. Statistical Data Analysis

The analytical data for the various yeast combinations were compared using ANOVA (α = 0.05, post-hoc Tukey test), Kruskal–Wallis test (α = 0.05, Dunn multiple comparison test), Principal Component Analysis (PCA) (Spearman correlation matrix), Hierarchical Cluster Analysis (HCA) (Euclidean distances), and were performed using XLSTAT 2017 (Addinsoft SARL, Paris, France).

To identify the volatile compounds likely contributing to the observed clusters, sorting task data were compared with the volatile compositions. Additionally, statistical analyses and Principal Component Analysis (PCA) were conducted using the XL-STAT software package (Addinsoft SARL, Paris, France). Hierarchical Cluster Analysis (HCA), as described by [39], was performed using XLSTAT 2017 (Addinsoft SARL, Paris, France).

## 3. Results and Discussion

In this study, for the first time, we applied commercial multi-starter inoculants combined with a fermentation temperature higher than the standard for white wine production, to evaluate the consistency of their inputs to the Lugana wine aromatic profile and obtain preliminary findings for a reduction of the demand for energy in the wine industry.

Several studies have reported positive effects of *M. pulcherrima*/*S. cerevisiae* sequential mixed fermentation on the varietal aroma and overall quality of white wines, such as Chardonnay, Verdicchio, Pecorino, and Sauvignon Blanc [20,42,43]. However, the selection of *M. pulcherrima* strains becomes a crucial factor, as different strains can significantly vary in their enzymatic activities and metabolite production, influencing both the aromatic profile and the dynamics of interaction with *S. cerevisiae* during fermentation.

### 3.1. Enzymatic Activity

The β-glucosidase and β-lyase activities were examined in the commercial yeast strains used in this study. The results, presented in Table 1, show that *M. pulcherrima* strains demonstrated remarkable activities (Figure S1), in accordance with literature data [16,22,44]. In contrast, *S. cerevisiae* strains exhibited only weak β-glucosidase activity, while they showed similar performance regarding β-lyase activity. These activities are associated with the potential enhancement of varietal aroma, particularly thiols, and the liberation of desirable aromas for white wine production [45,46]. This is of particular interest, given that not all *M. pulcherrima* strains can release volatile thiols [47]. While information on non-*Saccharomyces* yeasts’ ability to release volatile thiols in wine is limited, our results support the idea that *M. pulcherrima* might overcome nitrogen catabolic repression, which negatively affects β-lyase activity in *S. cerevisiae* [45].

### 3.2. Fermentation Kinetics and Performances

**Figure 1:**
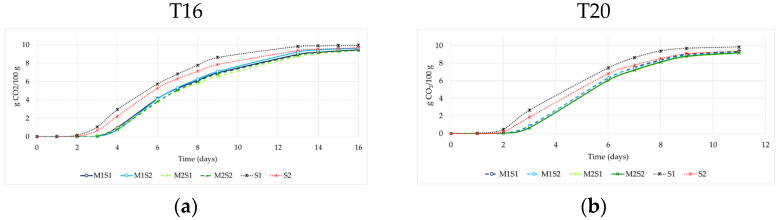
Fermentation kinetics of sequential cultures of *M. pulcherrima* M1 and M2 with *S. cerevisiae* S1 or S2, in natural Trebbiano grape must. Single cultures of *S. cerevisiae* S1 and S2 were inoculated at time 0 as controls. (**a**) Fermentation kinetics at 16 °C ± 1 °C (T16) (**b**) Fermentation kinetics at 20 °C ± 1 °C (T20).

The course of fermentations in the various trials, represented by CO_2_ release, is shown in Figure 1. Fermentations conducted at 20 °C ± 1 °C (T20), including both sequential inoculations and control trials, finished faster (within 11 days) compared to those at 16 °C ± 1 °C (T16), which concluded the process in 16 days. After a lag phase of two days, *S. cerevisiae* S1 showed the best performance at both temperatures, with a fermentation trend like that of *S. cerevisiae* S2. In contrast, no significant differences were observed in the fermentation kinetics among the trials using *M. pulcherrima* strains, with the four sequential inoculations clustering closely together, particularly in the T16 trials. However, in the T20 trials, the sequential inoculation of *M. pulcherrima* M2 with S2 exhibited the best kinetics during the final 8 days of fermentation. The shorter fermentation time at 20 °C than at 16 °C (reduction of approximately five days) is a finding particularly relevant, given that temperature control during fermentation is a major contributor to energy consumption in winemaking. Although direct energy measurements were not performed, our results align with previous studies [4,6] that demonstrate the potential for energy savings when using yeast strains capable of fermenting effectively at moderately elevated temperatures.

**Figure 2:**
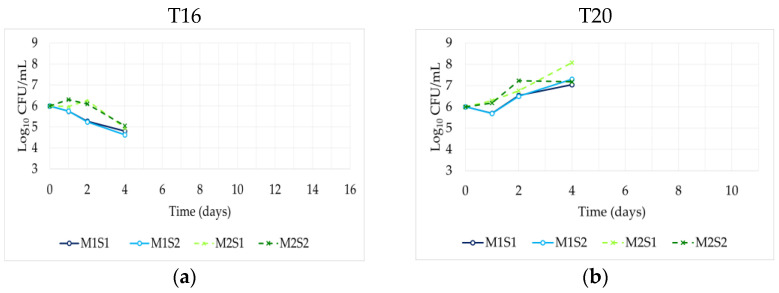

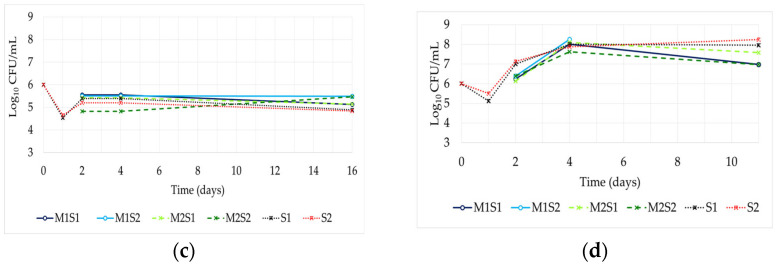
Yeast population dynamics during the sequential fermentation of *M. pulcherrima* M1 and M2 with *S. cerevisiae* S1 or S2 in natural Trebbiano grape must. Single cultures of *S. cerevisiae* S1 and S2 were inoculated at time 0 as controls. *M. pulcherrima* counts at 16 °C ± 1 °C (T16) (**a**) and 20 °C ± 1 °C (T20) (**b**). *S. cerevisiae* counts at 16 °C ± 1 °C (**c**) and 20 °C ± 1 °C (**d**).

The yeast population dynamics during fermentation, at the two temperatures, are shown in Figure 2. The native microbiota of the natural grape must was about 4 log CFU/mL before inoculation, and a heterogeneous yeast colony morphology was initially observed (Figure S2). However, after inoculation, this heterogeneity disappeared, suggesting that the inoculated yeasts dominated the fermentation process.

Figure 2a,b show the count of *M. pulcherrima* in the trials inoculated with the strains M1 and M2. In the T16 trials, an initial increase in cell number was observed only for M2, suggesting its better adaptability at low temperatures with respect to M1. In the T20 trials, the cell concentration of both *M. pulcherrima* strains increased, even after the inoculation of the *S. cerevisiae* strains, maintaining a high population until day 4. The M2S1 combination achieved the highest cell concentration, reaching 8 log CFU/mL at day 4, indicating superior growth performance under these conditions. After day 4, *M. pulcherrima* cells could not be detected at both temperatures due to their low tolerance to ethanol. These results suggest that *M. pulcherrima* may be especially effective as a multi-starter culture during the early stages of fermentation, promoting a quick onset of fermentation, particularly at higher temperatures. The role of non*-Saccharomyces* yeasts, such as *M. pulcherrima*, in the early stages of fermentation is further underscored, as their behaviour could be influenced by temperature management and inoculation timing [48]. This highlights the potential of combining *M. pulcherrima* as a sequential starter to accelerate fermentation and *S. cerevisiae* to complete it, maximizing the benefits of both yeasts in wine production.

The trend of the *S. cerevisiae* counts in the T16 and T20 trials is shown in Figure 2c and Figure 2d, respectively. After inoculating *S. cerevisiae* at 16 °C in control trials (S1 and S2), yeast cell counts initially declined, then recovered to approximately 5 log CFU/mL, maintaining stable levels through the end of fermentation. This behavior is consistent with previously observed population dynamics under similar winemaking conditions [49].

In the sequential fermentation trials, *S. cerevisiae,* inoculated after 48 h at 5 to 6 log CFU/mL levels, did not grow significantly, except for S2 in the mixed culture M2S2, maintaining similar final cell counts. Different dynamics were observed in the T20 trials. After an initial decline of the inoculated single strains S1 and S2 (from 6 to 5 log CFU/mL), a marked increase in cell counts was observed in all the trials until day 4, aligning with studies suggesting that higher temperatures promote faster growth rates and greater biomass production [50]. After that, the *S. cerevisiae* cell concentration remained stable in all the trials except for S2 in the mixed culture M1S2, showing a rapid decline after 4 days, likely due to competitive interactions or nutrient depletion.

These findings confirm the superior adaptability and fermentative performance of *S. cerevisiae* at 20 °C compared to 16 °C, where growth was generally more limited and variable. The trends observed in the sequential inoculation trials, particularly the lower growth following delayed inoculation at 16 °C, suggest that early *M. pulcherrima* activity, combined with environmental conditions, can significantly influence the development of *S. cerevisiae*. These results highlight the critical role of strain selection and temperature management in optimizing sequential fermentation strategies, by harnessing the early metabolic activity of *M. pulcherrima* while ensuring a robust completion of fermentation by *S. cerevisiae* [31].

### 3.3. Dominance of the Inoculated Strains

Molecular analyses confirmed the successful implantation of the commercial starter strains during the fermentation process of Lugana wine. Colonies randomly isolated from WL Nutrient Agar plates, showing the typical morphology of *M. pulcherrima* and *S. cerevisiae* [35], were subjected to DNA fingerprinting for confirmation at the strain level. An example of the genotypic profiles of *M. pulcherrima* isolates, obtained with the fingerprinting method described in the Section 2, is shown in Figure S3a. Comparison of the banding patterns of the inoculated commercial strains M1 and M2 with those of some isolates showed their correspondence; it indicated the dominance of the *M. pulcherrima* starters on the native non-*Saccharomyces* yeasts. Similarly, the Interdelta fingerprinting method applied to many *S. cerevisiae* isolates yielded profiles analogous to those of the inoculated commercial strains (Figure S3b), demonstrating the successful dominance and persistence of the starters through the fermentation process.

### 3.4. Chemical Analysis

The main characteristics of the Trebbiano grape must were: total acidity, 6.7 ± 0.3 g/L; initial sugar content, 192,92 ± 7.34 g/L; ammonium, 80.5 ± 4.4 mg/L; PAN, 114.4 ± 8.5 mg/L; YAN, 177.2 ± 9.8 mg/L; NTU, 290. Table 2 shows the chemical analysis of the wine fermented sequentially with *M. pulcherrima* and *S. cerevisiae* and the controls at the two temperatures.

For organizational reasons and to preserve the aromatic profile, fermentation was halted before completing sugar depletion. Residual sugar ranged from 0.60 g/L to 7.95 g/L, and strain S1 showed the highest sugar consumption. The chemical analysis of the wines revealed differences in most of the enological parameters analyzed, depending on the fermentation temperature and yeast inoculation strategy.

In agreement with previous studies [51,52], mixed fermentations resulted in a slight reduction in ethanol concentration compared to the controls. The lowest ethanol concentrations were observed in the wines produced with M1S2 at 20 °C (average 11.75% *v*/*v*) and M1S1 at 20 °C (11.81% *v*/*v*), suggesting a partial redirection of carbon metabolism toward alternative by-products [51,52].

Glycerol, a key compound influencing mouthfeel, remained stable across the trials, except for the M2S1 combination, which exhibited a lower concentration at both fermentation temperatures (around 3.32 g/L). While glycerol contributes to the smoothness and structure of wine [53,54], its increase is often linked to higher acetic acid production, which can negatively impact wine quality [54].

Regarding acetic acid, the highest concentration was observed in the control S2 at both temperatures (around 0.21 g/L). In contrast, the sequential inoculation of *M. pulcherrima* and *S. cerevisiae* resulted in an acetic acid concentration of around 0.12 g/L, representing a 33.33% decrease compared to the control’s value of around 0.18 g/L. In this study, acetic acid levels remained well below the sensory threshold of 0.80 g/L [55], with the highest value detected in the control (0.21 g/L). These findings support that *M. pulcherrima* can reduce volatile acidity when used in sequential fermentation, as previously reported [56].

On average, the total SO_2_ concentration, measured after fermentation, was lower in mixed fermentations compared to the control. The trial M2S1 showed the lowest values, with an average of 51.37 mg/L at 20 °C ± 1 °C and 56.53 mg/L at 16 °C—around 20 mg/L less than the control mean.

Additionally, at 16 °C, the M1S1 mixed fermentation was the only case in which acetaldehyde levels were lower than those of the respective control (S1); in all other cases, levels were equal to or higher than the corresponding controls. Nearly all other wines produced with *M. pulcherrima* strains showed acetaldehyde levels equal to or higher than those of the single inoculations with S1 and S2 in the T16 trials [51,57]. At 20 °C, sequential inoculations resulted in higher acetaldehyde concentrations than those observed at 16 °C, indicating that the effect on acetaldehyde formation is temperature dependent.

Mixed fermentations generally showed lower residual amino nitrogen levels than pure S. cerevisiae inoculations, at 16 °C and 20 °C. This suggests a higher nitrogen uptake in co-fermentations, likely due to the combined demand of the two yeast species [58]. Slightly higher residual values at 16 °C may reflect a reduced metabolic activity at lower temperatures.

Overall, these results confirm that the use of non-*Saccharomyces* yeasts can modulate wine composition, particularly in terms of ethanol reduction, volatile acidity control, and acetaldehyde formation.

### 3.5. Quantification of Volatile Compounds

The impact of the different inoculations was assessed using ANOVA (α = 0.05), conducted separately for each fermentation temperature (Table S1; [59,60,61,62,63,64]). Significant differences were observed in the wines fermented at 16 °C (T16) for total fatty acids, alcohols, branched-chain esters, C6 alcohols, and thiols. In contrast, in the wines fermented at 20 °C (T20), total ethyl esters, branched-chain esters, and thiols exhibited significant variation across inoculations. Furthermore, eleven individual volatile compounds showed statistically significant differences among the inoculation treatments in both T16 and T20 wines.

**Figure 3:**
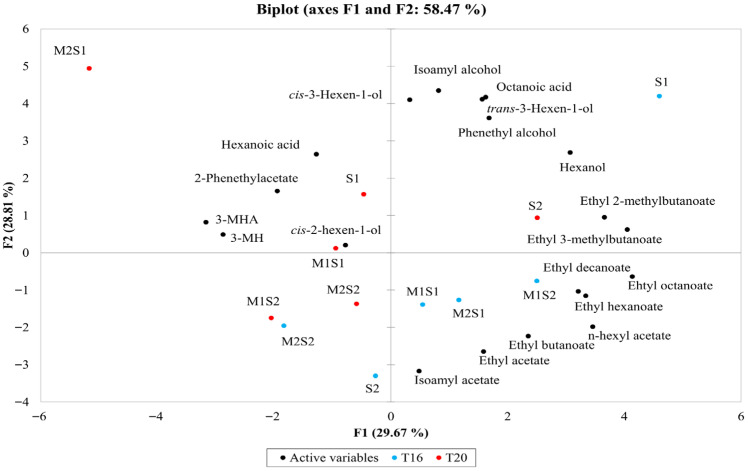
Principal Component Analysis (PCA) biplot showing the distribution of wine samples and volatile compounds along PC1, which reflects the inoculation strategy, while PC2 distinguishes the effects of temperature. The wines were fermented sequentially with *M. pulcherrima* M1 and M2 and *S. cerevisiae* S1 or S2 at 16 °C ± 1 °C (T16) and 20 °C ± 1 °C (T20). Single cultures of *S. cerevisiae* S1 and S2 were inoculated at time 0 as controls.

The principal component analysis (PCA) to assess the overall distribution of volatile profiles is shown in Figure 3.

The first two principal components accounted for 58.7% of the total variance, with PC1 accounting for 29.67% and PC2 accounting for 28.81%. PC1 primarily reflected the effect of different inoculation strategies, separating samples based on their yeast combinations. Specifically, M1S1 and S1 at 20 °C clustered at positive values, while S2 and M2S2 at 16 °C were positioned at negative values. This distribution suggests that the inoculation strategy influences the balance between esters and higher alcohols/acids, as indicated by the positioning of volatile compounds along this axis. Conversely, PC2 was strongly associated with fermentation temperature, distinguishing T16 from T20 samples. Notably, the S1 T20 sample clustered closer to the T16 group, exhibiting high positive loadings on PC1. The variability within each temperature condition, attributable to different inoculation strategies, was mainly captured by PC1. However, the distinct clustering patterns within each temperature group suggest a complex interaction between fermentation temperature and inoculation type in shaping the volatile composition of the wines.

**Figure 4:**
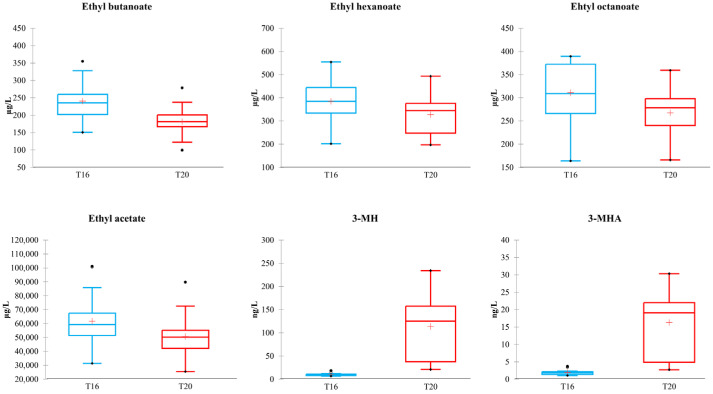
Content of significantly different compounds (Kruskal–Wallis, α = 0.05) between wines obtained at 16 °C ± 1 °C (T16) and 20 °C ± 1 °C (T20). Units of measurement are µg/L for ethyl butanoate, ethyl octanoate, ethyl hexanoate, and ethyl acetate; ng/L for 3-MH and 3-MHA.

Significant variations in compound levels were observed with temperature changes, with some compounds increasing (3-MHA) and others decreasing (ethyl butanoate) at higher temperatures. The results of temperature effects on the volatile chemical profile are reported in Figure 4, where major variations were observed for esters and potent odorants such as thiols.

Our results confirm that lower fermentation temperatures (16 °C) lead to slower yet more stable fermentations and promote the synthesis of esters and other volatile compounds associated with fruity aromas, as reported by [10,65]. In contrast, fermentations conducted at higher temperatures (20 °C) were faster but showed increased production of volatile thiols, particularly 3-mercaptohexanol (3-MH) and 3-mercaptohexyl acetate (3-MHA), corroborating findings by [46,66], who demonstrated a positive correlation between temperature and thiol content. Nevertheless, elevated temperatures may induce metabolic stress in yeast cells, potentially compromising the aromatic complexity of the final wine, as noted by [67].

Lower temperatures favored the synthesis of ethyl esters and ethyl acetate, although the differences were moderate, ranging from a 14% increase for ethyl octanoate to 24% for ethyl butanoate. These esters are pleasant fermentative compounds contributing to fruity aromas [68]. Low molecular weight ethyl esters such as ethyl butanoate originate from α-ketobutanoate [69], whereas medium- to long-chain fatty acid ethyl esters are synthesized during early lipid biosynthesis via esterification of acyl-CoA intermediates [70]. In agreement with our findings, several studies have reported that low fermentation temperatures enhance ester concentrations by modulating yeast metabolism and reducing volatilization of these compounds [10,65,71,72,73].

Conversely, thiol production was strongly enhanced at higher fermentation temperatures. Both 3-MH and 3-MHA exhibited concentrations approximately ten times higher in T20 wines than those at 16 °C. Although substantial variability was observed among T20 wines, this aspect is further examined in the following section. Similar results were reported by [66] and [46], despite differences in experimental conditions (13 °C vs. 20 °C; 18 °C vs. 28 °C). Thiols, which contribute to desirable aromas such as boxwood, grapefruit, and passion fruit, are characteristic of Sauvignon Blanc wines [74,75] and have odor thresholds in the ng/L range [64]. Notably, thiol concentrations in T16 wines remained below odor thresholds, whereas in T20 wines they often exceeded these thresholds, likely enhancing their aromatic profiles.

Although lower fermentation temperatures are generally associated with enhanced fruity ester production, our results demonstrate that higher temperatures lead to a modest decrease in fruity esters but a substantial increase in thiols—compounds responsible for distinct tropical and citrus aromas [76].

**Figure 5:**
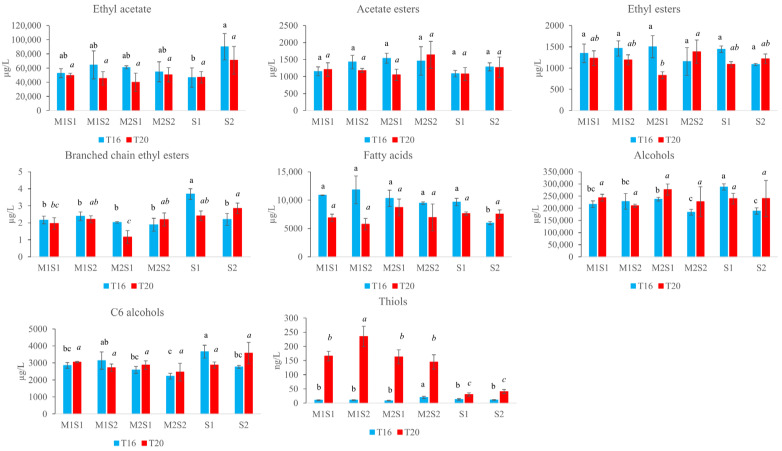
Total concentration of eight biochemical classes showing significant variation across treatments. The wines were fermented sequentially with *M. pulcherrima* M1 and M2 and *S. cerevisiae* S1 or S2 at 16 °C ± 1 °C (T16) and 20 °C ± 1 °C (T20). Single cultures of *S. cerevisiae* S1 and S2 were inoculated at time 0 as controls. One-way ANOVA was used to compare data. Different letters in a column represent significantly (*p* ≤ 0.05) different concentrations expressed in milligrams per liter; regular font letters refer to the T16 samples, while italicized letters refer to the T20 samples. Standard errors were used for the error bars.

The total content of volatile compounds, according to different biochemical classes, is shown in Figure 5.

Thiols were significantly influenced by inoculation strategy in both T16 and T20 wines. At T16, the highest thiol concentrations were found in wines produced with M2S2 and S1 inoculations. At T20, sequential inoculations—especially M1S2—resulted in significantly higher thiol levels compared to wines fermented with *S. cerevisiae* alone, with increases ranging from two- to sixfold. Although *M. pulcherrima* is generally associated with enhanced thiol release compared to *S. cerevisiae* [19,20,47], some studies have reported contrary findings [77]. In our study, improved thiol release efficiency by sequential inoculation was observed exclusively at 20 °C.

Regarding ester production, only ethyl acetate was significantly influenced by inoculation at 16 °C. However, sequential inoculations showed levels comparable to *S. cerevisiae*, with significant differences observed only among the *S. cerevisiae* strains. Among acetate esters, only n-hexyl acetate in T20 wines exhibited significant differences, with S2 and M1S2 being distinct (Table S1). Ethyl ester levels in T16 wines did not vary significantly among inoculations; whereas in T20 wines these compounds were lower in M2S1 than in M2S2, and were not statistically different from those in the respective control.

Branched-chain esters showed significant differences among inoculation strategies within each temperature. At T16, S1 exhibited the highest concentrations of these esters, while sequential inoculations were similar to S2. In T20 wines, M1S2 and M2S2 resembled S1 and S2. Conversely, M1S1 showed similar content only to S1 and M2S1 showed lower levels compared to both S1 and S2. These esters, known for their red fruit aromas and low odor thresholds (3–18 μg/L), originate from the esterification of branched-chain fatty acids derived from amino acid metabolism [78]. In this study, their concentrations remained below their respective thresholds.

Fatty acids and higher alcohols also varied significantly with inoculation in T16 wines. Sequential inoculations yielded fatty acid levels comparable to S1 but higher than S2. Straight-chain fatty acids are by-products of fatty acid synthesis (C16 and C18) from saturated fats and generally have a negative effect on wine aroma [79]. Although *M. pulcherrima* is often associated with increased fatty acid production [20,51], results across studies are inconsistent.

Regarding higher alcohols, sequential inoculations with S1 showed lower levels than S1 alone, whereas inoculations with S2 produced similar or slightly higher levels than S2. These compounds are formed via the Ehrlich pathway during amino acid catabolism and have mixed impacts on wine aroma, most of which are negative, except phenethyl alcohol [76]. Literature reports both increases (up to 33%) and decreases (up to 30%) in higher alcohols during sequential fermentations [19]; in our case, differences were relatively minor.

Finally, C6 alcohols, including hexanol and cis-2-hexen-1-ol, were significantly affected by inoculation (Figure 5 and Table S1). M1S1 and M2S1 at T16 showed lower total C6 alcohols compared to S2; conversely, M1S2 and M2S2 showed similar content to S2. Considering individual C6 alcohols, in T16 wines, M1S1, M2S1 had lower hexanol levels than their *S. cerevisiae* controls, whereas in T20, M1S2 and M2S2 exhibited lower levels than S2. C6 alcohols, associated with green and grassy aromas, are generally considered undesirable [61]. Previous studies have reported lower C6 alcohol production in co-inoculations of *M. pulcherrima* and *S. cerevisiae* compared to *S. cerevisiae* alone.

### 3.6. Sensory Evaluation

**Figure 6:**
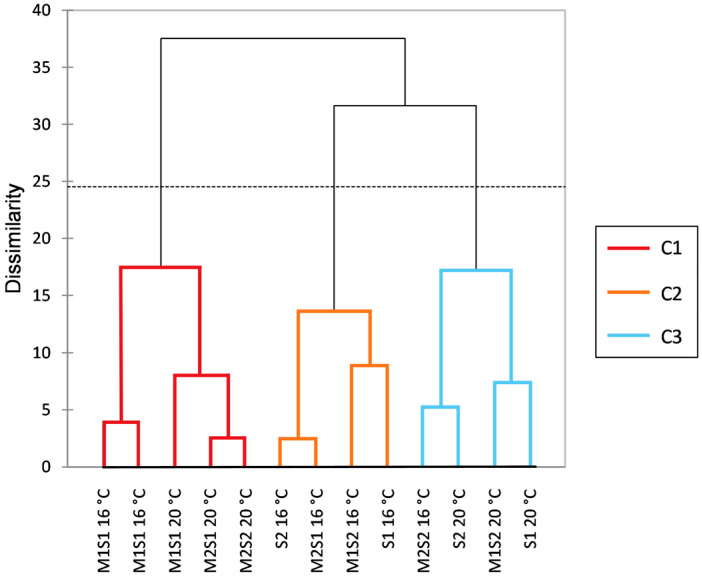
Hierarchical cluster analysis (HCA) of the sorting task data for the 12 wines analyzed, including the technical replicate (M1S1 at 16 °C ± 1 °C). The wines were fermented sequentially with *M. pulcherrima* M1 and M2 and *S. cerevisiae* S1 or S2 at 16 °C ± 1 °C and 20 °C ± 1 °C. Single cultures of *S. cerevisiae* S1 and S2 were inoculated at time 0 as controls.

An essential element of characterizing volatile profiles is their impact on the perceived aroma of wines. To explore this relationship, a sensory evaluation of the wines was conducted. The sensory data were subjected to hierarchical cluster analysis (HCA), and the results are shown in Figure 6.

The reproducibility of the panel was demonstrated by the clustering of the two replicated samples with minimal dissimilarity within the same cluster. The dendrogram identified three main clusters. The first cluster (C1) comprised four wines, all from sequential fermentations predominantly carried out at 20 °C, with only one sample at 16 °C. The second cluster (C2) consisted of wines fermented at 16 °C with *S. cerevisiae* alone and sequential fermentations involving *M. pulcherrima*. The third cluster (C3) was mainly formed by T20 and M2S2 T16 wines. It was observed that fermentation temperature played a key role in the clustering of wines, although some samples were distributed across different clusters.

**Figure 7:**
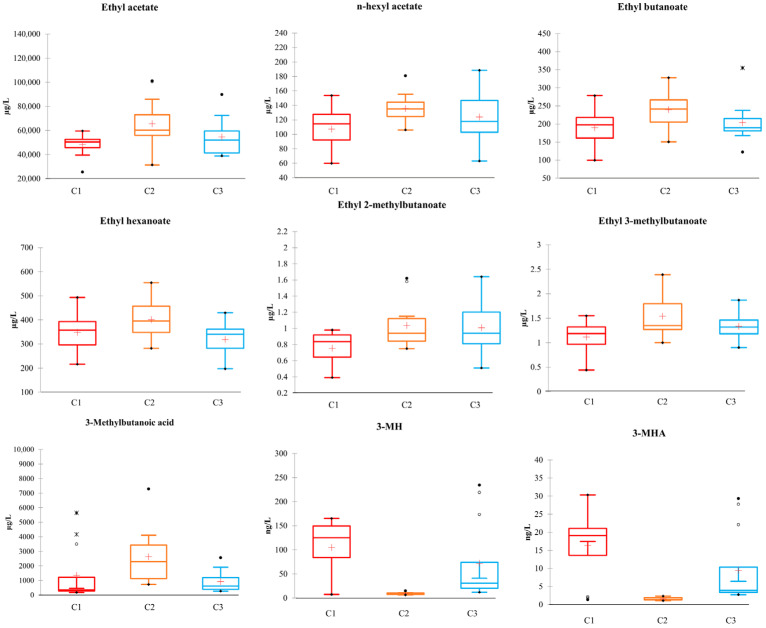
Box plots of significantly different compounds (Kruskal–Wallis, α = 0.05) between the sensory clusters C1, C2, and C3. Units of measurement are µg/L for all compounds except 3-MH and 3-MHA (ng/L).

Sorting task data were compared with volatile compositions to identify the volatile compounds most likely contributing to the observed clusters. Volatile chemical data were submitted to the Kruskal–Wallis analysis. Globally, nine compounds showed a significantly different content between clusters (Figure 7). C1 differed from C2 for lower ethyl acetate, n-hexyl acetate and ethyl butanoate content, while C3 showed intermediate levels. As for branched esters, C1 showed lower contents than both C2 and C3, which, in contrast, exhibited similar levels. A different pattern was shown by ethyl hexanoate and 3-methylbutanoic acid, which differentiated C2 from C3, while C1 showed intermediate levels. The most significant differences were observed about the thiols, 3-MH and 3-MHA, which showed a content close to zero for C2, which consisted only of T16 wines, while much higher contents, albeit with significant variability for C1 and C3. Notably, in C1 and C3, both thiols were often above the odor threshold (OT), while in C2, they consistently remained below it.

## 4. Conclusions

Although the use of mixed or multi-starter cultures has been widely investigated in recent years, particularly for enhancing aroma complexity in white wines, our study provides a novel contribution by combining commercial *M. pulcherrima* strains with *S. cerevisiae* under controlled temperature conditions (16 °C and 20 °C), specifically in Lugana wine production. This approach allowed us to assess not only the aromatic impact but also fermentation kinetics and potential energy savings, aspects that are less explored in previous literature. The results highlight the importance of optimizing key parameters—particularly yeast strain selection and temperature management—to enhance volatile compound production while supporting process sustainability. The sequential inoculation effectively enriched the wine with varietal thiols and tropical aroma markers, especially at 20 °C. Among the tested combinations, M2S2 and M1S2 yielded the most promising aromatic profiles, confirming the potential of *M. pulcherrima* as a non-*Saccharomyces* partner in early fermentation stages.

These findings provide practical insights for the wine industry by supporting the use of controlled sequential strategies at moderately elevated temperatures as a viable approach to enhance both aromatic complexity and energy efficiency in white wine production. Further studies should investigate the sensory outcomes of these chemical shifts and assess their applicability on an industrial scale.

## Figures and Tables

**Table 1:** Distinctive enological features of the commercial yeasts used in this study. Data were obtained from technical datasheets and from the results obtained here.

Code	Species	Commercial Name	Producer	Temperature Range (°C)	*β*-Lyase Activity ^a^	*β*-Glucosidase Activity ^a^	Note	Reference
M1	*M. pulcherrima*	Level^2^ Flavia	Lallemand ^b^	15–22	+ ^c^	+	Release thiols for fruity aromas and, with *S. cerevisiae*, boosts wine aromas during fermentation.	[31]
M2	*M. pulcherrima*	Level^2^ Initia	Lallemand	4–18	+	+	Yeast prevents oxidation, reducing copper, and minimizing SO_2_ use, preserves aromas.	[32]
S1	*S. cerevisiae*	EC 1118	Lallemand	10–30	+	-+ ^d^	Reference yeast for base and sparkling wines.	[31]
S2	*S. cerevisiae*	Zymaflore X5	Laffort ^e^	13-nr ^f^	+	-+	Yeast for white and rosé wines with high aromatic intensity.	[33]

^a^ Enzymatic activities evaluated in this study; ^b^ Lallemand Inc., Castel d’Azzano, Italy; ^c^ positive enzymatic activity; ^d^ weak enzymatic activity; ^e^ Laffort, Bordeaux, France; ^f^ not reported.

**Table 2:** Chemical analysis of the wine fermented sequentially with *M. pulcherrima* M1 and M2 and *S. cerevisiae* S1 or S2 at two temperatures (16 °C ± 1 °C and 20 °C ± 1 °C). Single cultures of *S. cerevisiae* S1 and S2 were inoculated at time 0 as controls. The data are the means of two analytical replicates.

Yeast Combination	Enological Parameters							
	Residual Sugars (g/L)	Ethanol (% *v*/*v*)	Glycerol (g/L)	Total Acidity (g/L)	Acetic Acid (g/L)	Total Sulfite (mg/L)	Acetaldehyde (mg/L)	Amino Nitrogen (mg/L)
Trial at 16 °C								
M1S1	4.93 ± 0.54	11.99	4.93 ± 0.38	10.54 ± 0.31	0.16 ± 0.02	81.02 ± 6.97	19 ± 1.79	6 ± 0.8
M1S2	3.49 ± 0.21	12.07	4.58 ± 0.43	10.1 ± 0.22	0.13 ± 0.01	67.87 ± 5.4	19 ± 1.37	7 ± 0.7
M2S1	4.08 ± 0.4	12.09	3.4 ± 0.25	10.58 ± 0.24	0.11 ± 0.02	56.53 ± 6.78	23 ± 3.43	6 ± 0.5
M2S2	4.07 ± 0.31	11.96	4.63 ± 0.39	11.66 ± 0.34	0.15 ± 0.01	53.54 ± 5.71	17 ± 1.89	8 ± 0.7
S1	0.6 ± 0.06	12.08	4.88 ± 0.37	9.73 ± 0.29	0.17 ± 0.02	67.38 ± 4.04	23 ± 1.96	8 ± 0.8
S2	2.6 ± 0.18	12.00	4.83 ± 0.3	10.22 ± 0.51	0.2 ± 0.02	86.15 ± 6.66	14 ± 1.48	9 ± 1.1
Trial at 20 °C								
M1S1	5.96 ± 0.76	11.81	5.33 ± 0.38	7.88 ± 0.24	0.11 ± 0.01	76.1 ± 3.93	13 ± 1.24	6 ± 0.5
M1S2	6.01 ± 0.8	11.75	5.06 ± 0.45	10.21 ± 0.35	0.12 ± 0.01	74.14 ± 5.78	14 ± 1.39	6 ± 0.4
M2S1	7.95 ± 0.59	11.82	3.23 ± 0.4	11.17 ± 0.44	0.11 ± 0.01	51.37 ± 5.55	9 ± 0.72	6 ± 0.7
M2S2	7.41 ± 0.84	11.88	5.08 ± 0.46	12.02 ± 0.46	0.11 ± 0.01	74.99 ± 4.92	16 ± 1.84	7 ± 0.8
S1	1.81 ± 0.17	12.18	4.07 ± 0.44	10.98 ± 0.48	0.12 ± 0.02	83.74 ± 4.12	18 ± 1.26	6 ± 0.9
S2	6.52 ± 0.37	12.08	5.09 ± 0.51	11.71 ± 0.66	0.23 ± 0.02	69.59 ± 4.15	19 ± 2.68	8 ± 0.7

## Data Availability

The original contributions presented in the study are included in the article/Supplementary Material; further inquiries can be directed to the corresponding author.

## Supplementary Materials

The following supporting information can be downloaded at: https://www.mdpi.com/article/10.3390/foods14203538/s1. Figure S1. β-glucosidase (a) and β-lyase (b) enzymatic activities of commercial starter strains used in this study. Figure S2. Morphologies of the native yeast colonies detected in natural grape must on WL Nutrient agar medium plate. Figure S3. Examples of genotypic profiles of commercial starter strains and yeast isolates picked from WL Nutrient Agar medium plates during the fermentation process. (a) fingerprinting with the combination of (GTG)_5_ and M13 primers of *M. pulcherrima* M1 (Level^2^ Flavia) and M2 (Level^2^ Initia), lines 2–6 and 8–12, new isolates from mixed fermentations in different conditions at various time points; (b) fingerprinting using the Interdelta fingerprinting method of *S. cerevisiae* S1 (Zymaflore X5) and S2 (EC 1118), lines 2–6 and 8–12, new isolates from mixed fermentations in different conditions at various time points. M: Molecular weight marker (100–10,000 bp) GeneRuler™ DNA Ladder Mix (Thermo Scientific). Table S1. Concentration (μg/L) of volatile compounds in the wines fermented sequentially with *M. pulcherrima* and *S. cerevisiae* strains. Pure cultures of *S. cerevisiae* were used as controls.
