# Supplementary material for: Influence of Fermentation Temperature and Metschnikowia pulcherrima/Saccharomyces cerevisiae Multi-Starter Cultures on the Volatile Compounds of Lugana Wine
Source: Foods. 2025 Oct 17;14(20):3538. doi: 10.3390/foods14203538 (PMC12562818; doi:10.3390/foods14203538)
Supplement: Supplementary file 1 [file foods-14-03538-s001.zip › foods-3900727-supplementary.pdf]

Supplementary Material

Influence of Fermentation Temperature and *Metschnikowia pulcherrima*/*Saccharomyces cerevisiae* Multi-Starter Cultures on the Volatile Compounds of Lugana Wine

Giulia Bertazzoli [1,†], Emma Pelizza [1,†], Giovanni Luzzini [1], Giovanna E. Felis [1,2], Maurizio Ugliano [1] and Sandra Torriani [1,*]

[1] Department of Biotechnology, University of Verona, 37134 Verona, Italy; giulia.bertazzoli@univr.it (G.B.); emma.pelizza@univr.it (E.P.); giovanni.luzzini@univr.it (G.L.); giovanna.felis@univr.it (G.E.F.); maurizio.ugliano@univr.it (M.U.)
[2] VUCC-DBT, Verona University Culture Collection, University of Verona, 37134 Verona, Italy
[*] Correspondence: sandra.torriani@univr.it
[†] These authors contributed equally to this work.

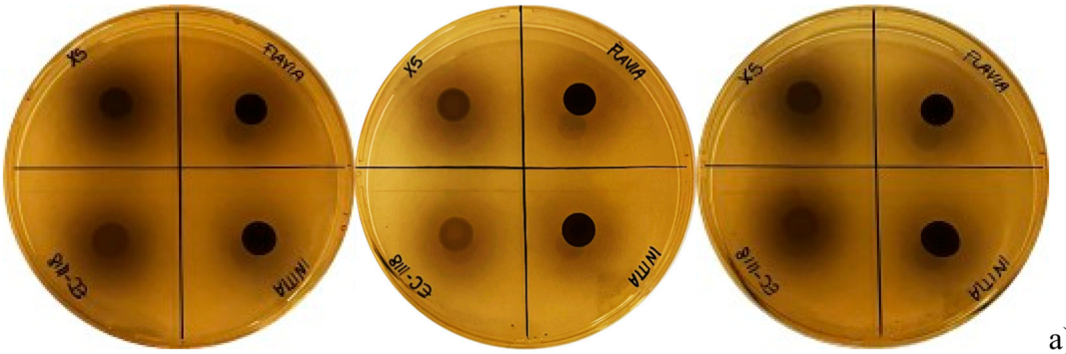

a)

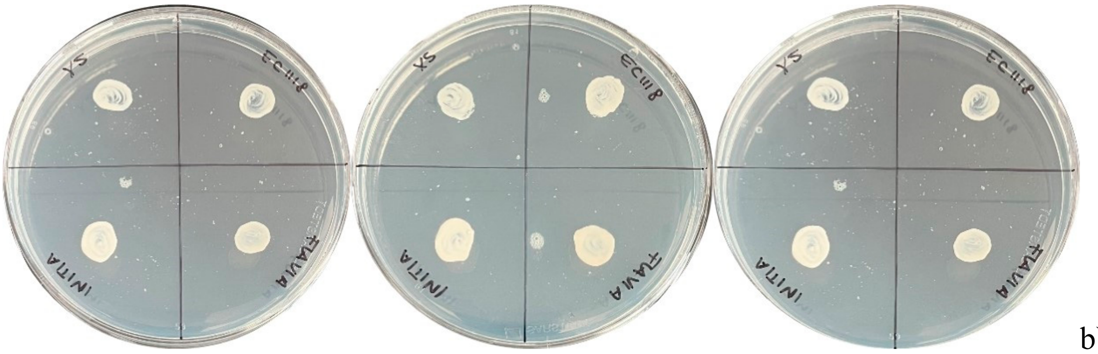

b)

Figure S1. β-glucosidase (a) and β-lyase (b) enzymatic activities of the commercial starter strains used in this study.

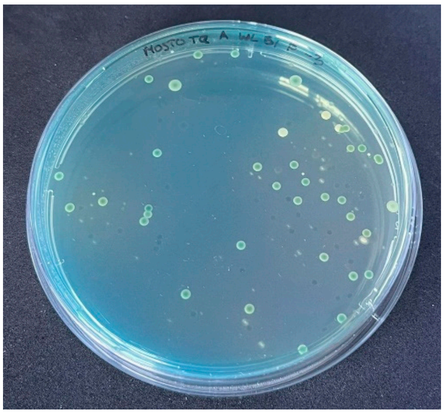

Figure S2. Morphologies of the native yeast colonies detected in natural grape must on WL Nutrient agar medium plate.

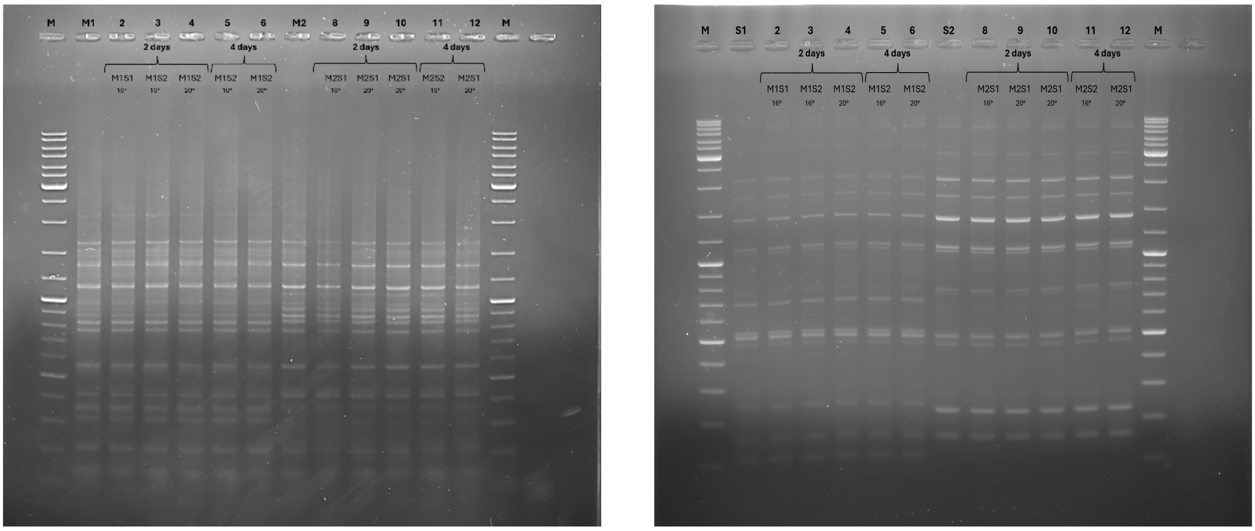

a) b)

Figure S3. Examples of genotypic profiles of commercial starter strains and yeast isolates picked from WL Nutrient Agar medium plates during the fermentation process.

(a) fingerprinting with the combination of $(GTG)_5$ and M13 primers of *M. pulcherrima* M1 (Level[2] Flavia) and M2 (Level[2] Initia), lines 2-6 and 8-12, new isolates from mixed fermentations in different conditions at various time points;

(b) fingerprinting using the Interdelta fingerprinting method of *S. cerevisiae* S1 (Zymaflore X5) and S2 (EC 1118), lines 2-6 and 8-12, new isolates from mixed fermentations in different conditions at various time points.

M: Molecular weight marker (100–10,000 bp) GeneRuler™ DNA Ladder Mix (Thermo Scientific).

Table S1. Concentration (μg/L) of volatile compounds in the wines fermented sequentially with *M. pulcherrima* and *S. cerevisiae* strains. Pure cultures of *S. cerevisiae* were used as controls.

Volatile compound	T16							T20							Odor threshold (μg/L)	OT reference	OD
	M1S1	M1S2	M2S1	M2S2	S1	S2	Sign.(1)	M1S1	M1S2	M2S1	M2S2	S1	S2	Sign. (1)			
**Acetate esters**																	
Ethyl acetate	52746 ± 6415$^{ab(2)}$	64487 ± 19728^{ab}	61044 ± 2086^{ab}	54682 ± 14121^{ab}	46631 ± 13653^{b}	90151 ± 18544^{a}	+	49798 ± 2793^{a}	45490 ± 9408^{a}	40022 ± 12866^{a}	50517 ± 10134^{a}	47082 ± 7995^{a}	70965 ± 19612^{a}	-	12264	[59]	Sweet-fruity, pear, pineapple, solvent
Isoamyl acetate	919 ± 122^{a}	1151 ± 180^{a}	1242 ± 128^{a}	1166 ± 386^{a}	825 ± 80^{a}	1019 ± 119^{a}	-	969 ± 149^{a}	960 ± 57^{a}	783 ± 174^{a}	1328 ± 305^{a}	814 ± 188^{a}	951 ± 282^{a}	-	30	[60]	Banana, fruity, sweet
2-Phenethylacetate	121 ± 21^{a}	126 ± 26^{a}	168 ± 38^{a}	174 ± 22^{a}	134 ± 6^{a}	130 ± 8^{a}	-	113 ± 47^{a}	109 ± 21^{a}	204 ± 18^{a}	189 ± 65^{a}	169 ± 18^{a}	149 ± 27^{a}	-	250	[60]	Rose, honey, and mild fruitiness
n-hexyl acetate	115 ± 14^{a}	150 ± 33^{a}	128 ± 20^{a}	121 ± 21^{a}	128 ± 15^{a}	137 ± 8^{a}	-	125 ± 5^{ab}	111 ± 7^{bc}	65.3 ± 5.4^{c}	123 ± 28^{ab}	92.0 ± 27.8^{bc}	171 ± 15^{a}	+	1500	[59]	Apple, cherry, pear, floral
**Ethyl esters**																	
Ethyl butanoate	224 ± 23^{a}	281 ± 40^{a}	278 ± 40^{a}	260 ± 85^{a}	217 ± 26^{a}	183 ± 35^{a}	-	180 ± 31^{a}	192 ± 15^{a}	133 ± 34^{a}	222 ± 54^{a}	176 ± 58^{a}	187 ± 10^{a}	-	20	[60]	Pineapple, apple, and banana
Ethyl hexanoate	378 ± 67^{a}	409 ± 63^{a}	453 ± 90^{a}	326 ± 115^{a}	446 ± 39^{a}	298 ± 14^{a}	-	374 ± 5^{ab}	343 ± 23^{ab}	223 ± 7^{b}	419 ± 90^{a}	296 ± 97^{ab}	313 ± 77^{ab}	+	14	[61]	Strawberry, anise, green apple, brandy
Ehtyl octanoate	329 ± 55^{a}	356 ± 53^{a}	324 ± 48^{a}	248 ± 79^{a}	359 ± 18^{a}	250 ± 7^{a}	-	279 ± 37^{a}	263 ± 34^{ab}	179 ± 20^{b}	313 ± 66^{a}	281 ± 7^{a}	291 ± 20^{a}	+	5	[61]	fFtty, sweet, floral, pear, pineapple
Ethyl decanoate	417 ± 87^{a}	416 ± 44^{a}	446 ± 96^{a}	321 ± 53^{a}	422 ± 16^{a}	354 ± 21^{a}	-	400 ± 120^{a}	397 ± 66^{a}	296 ± 23^{a}	433 ± 82^{a}	334 ± 69^{a}	427 ± 22^{a}	-	200	[61]	Waxy, fruity, apple, grape
**Branched chain esters**																	
Ethyl 2-methylbutanoate	0.91 ± 0.09^{b}	0.91 ± 0.08^{b}	0.81 ± 0.05^{b}	0.68 ± 0.19^{b}	1.45 ± 0.26^{a}	0.97 ± 0.16^{b}	+	0.82 ± 0.1^{bc}	0.84 ± 0.05^{bc}	0.45 ± 0.1^{c}	0.84 ± 0.14^{bc}	1.31 ± 0.33^{a}	1.21 ± 0.1^{ab}	+	18	[61]	Apple, strawberry, tropical fruit
Ethyl 3-methylbutanoate	1.25 ± 0.14^{b}	1.48 ± 0.17^{b}	1.22 ± 0.07^{b}	1.21 ± 0.23^{b}	2.24 ± 0.13^{a}	1.23 ± 0.2^{b}	+	1.14 ± 0.24^{ab}	1.37 ± 0.16^{a}	0.71 ± 0.28^{b}	1.36 ± 0.24^{ab}	1.11 ± 0.22^{ab}	1.64 ± 0.27^{a}	+	3	[62]	Fruity, apple, sweetish
**Fatty acid**																	
Hexanoic acid	3277 ± 606^{ab}	3555 ± 433^{ab}	3964 ± 545^{a}	4210 ± 389^{a}	3852 ± 53^{a}	2490 ± 58^{b}	+	3225 ± 466^{a}	2845 ± 546^{a}	4690 ± 957^{a}	3613 ± 965^{a}	3393 ± 70^{a}	3018 ± 66^{a}	-	420	[61]	Cheese, sweaty, fatty
Octanoic acid	3181 ± 463^{ab}	3470 ± 344^{b}	3062 ± 188^{ab}	3185 ± 282^{ab}	4241 ± 492^{a}	2621 ± 224^{ab}	+	3311 ± 90^{a}	2606 ± 407^{a}	3758 ± 549^{a}	3037 ± 1314^{a}	3806 ± 148^{a}	3675 ± 861^{a}	-	500	[61]	Fatty, rancid, cheese
**Alcohols**																	
Isoamyl alcohol	205519 ± 15456^{bc}	215087 ± 29779^{bc}	223675 ± 7836^{ab}	172710 ± 10653^{c}	267713 ± 10683^{a}	178066 ± 13741^{c}	+	230230 ± 15189^{a}	200216 ± 3745^{a}	261139 ± 21452^{a}	213347 ± 57150^{a}	222989 ± 19588^{a}	225902 ± 69902^{a}	-	30000	[60]	Fruity and banana-like notes
Phenethyl alcohol	11043 ± 2023^{b}	13039 ± 2894^{b}	13548 ± 2173^{b}	11357 ± 1043^{b}	20145 ± 2306^{a}	10824 ± 651^{b}	+	13328 ± 1142^{a}	10172 ± 2519^{a}	16075 ± 2034^{a}	14044 ± 4089^{a}	16513 ± 3084^{a}	14233 ± 4516^{a}	-	14000	[61]	Rose and honey, with subtle spicy and sweet

**C_6 alcohols**																	
Hexanol	2434 ± 148^{bc}	2656 ± 446^{ab}	2212 ± 172^{bc}	1879 ± 146^{c}	3315 ± 334^{a}	2473 ± 76^{bc}	+	2558 ± 43^{ab}	2315 ± 233^{b}	2430 ± 219^{ab}	2091 ± 442^{b}	2604 ± 184^{ab}	3215 ± 596^{a}	+	8000	[61]	Herbaceous, grass, woody, toasty, dry
trans-3-Hexen-1-ol	32.1 ± 2.5^{a}	40.8 ± 11.9^{a}	34.2 ± 3.7^{a}	31.1 ± 3.2^{a}	41.5 ± 5.4^{a}	31.9 ± 1.4^{a}	-	34.9 ± 0.8^{a}	32.0 ± 2.1^{a}	41.0 ± 5.7^{a}	30.8 ± 5.8^{a}	34.3 ± 3.7^{a}	41.5 ± 5.6^{a}	-	1000	[63]	Grass, leaves, green vegetables
cis-3-Hexen-1-ol	20.3 ± 2.1^{a}	21.4 ± 4.1^{a}	22.1 ± 3.86^{a}	20.3 ± 1.5^{a}	24.6 ± 1.2^{a}	18.5 ± 0.7^{a}	-	21.1 ± 0.7^{a}	19.7 ± 2.1^{a}	25.4 ± 3.4^{a}	20.5 ± 4.2^{a}	19.7 ± 1.6^{a}	22.1 ± 1.6^{a}	-	400	[60]	Grass, green leaves, cucumber
cis-2-hexen-1-ol	367 ± 19^{ab}	420 ± 60^{a}	325 ± 28^{abc}	289 ±38^{bc}	282 ± 50^{bc}	242 ± 11^{c}	+	437 ± 5^{a}	353 ± 46^{ab}	379 ± 26^{ab}	320 ± 68^{bc}	218 ± 14^{c}	297 ± 28^{bc}	+	10000	[63]	Grass, green vegetables, herbs
**Thiols (ng/L)**																	
3-MH	8.47 ± 0.89^{b}	8.39 ± 1.32^{b}	7.03 ± 0.71^{b}	16.3 ± 3.6^{a}	11.8 ± 2.5^{ab}	9.69 ± 1.34^{b}	+	144 ± 16^{b}	208 ± 32^{a}	139 ± 22^{b}	126 ± 23^{b}	27.3 ± 5.6^{c}	35.4 ± 6.3^{c}	+	0,06	[64]	Grapefruit, passion fruit, fresh, exotic fruits
3-MEA	1.77 ± 0.41^{b}	2.15 ± 0.17^{b}	1.4 ± 0.35^{b}	3.55 ± 0.14^{a}	1.43 ± 0.42^{bc}	1.35 ± 0.11^{b}	+	21 ± 0.7^{b}	26.3 ± 3.8^{a}	24.0 ± 6.46^{a}	18.6 ± 1.65^{b}	2.9 ± 0.24^{c}	4.93 ± 1.29^{c}	+	0,0042	[64]	Tropical fruit, citrus, and grapefruit

Mean ± standard deviation values of three independent replicates are indicated;

(1) Sign. is short for significativity according to ANOVA (α=0.05); + = significant value; - = not significant value.

(2) Lowcase letters refers to different group according to Tukey post-hoc test;

OT = Odor Threshold.

OD = Odor Descriptor.

References

59. Maarse, H. *Volatile Compounds in Foods and Beverages*; Maarse, H., Ed.; 1st ed.; Routledge, New York, NY, USA, 2017; ISBN 978-0-203-73428-5.
60. Guth, H. Quantitation and Sensory Studies of Character Impact Odorants of Different White Wine Varieties. *J. Agric. Food Chem.* 1997, *45*, 3027–3032, doi:10.1021/jf970280a.
61. Wildenradt, H.L.; Christensen, E.N.; Stackler, B.; Caputi, A.; Slinkard, K.; Scutt, K. Volatile Constituents of Grape Leaves. I. Vitis Vinifera Variety "Chenin Blanc." *Am J Enol Vitic.* 1975, *26*, 148–153, doi:10.5344/ajev.1975.26.3.148.
62. Ferreira, L.; Perestrelo, R.; Camara, J. Comparative Analysis of the Volatile Fraction from Annona Cherimola Mill. Cultivars by Solid-Phase Microextraction and Gas Chromatography–Quadrupole Mass Spectrometry Detection. *Talanta* 2009, *77*, 1087–1096, doi:10.1016/j.talanta.2008.08.011.
63. Ferreira, V.; Lopez, R.; Cacho, J.F. Quantitative Determination of the Odorants of Young Red Wines from Different Grape Varieties. *J. Sci. Food Agric.* **2000**, *80*, 1659–1667. https://doi.org/10.1002/1097-0010(20000901)80:11<1659::AID-JSFA693>3.0.CO;2-6.
64. Tominaga, T.; Baltenweck-Guyot, R.; Gachons, C.P.D.; Dubourdieu, D. Contribution of Volatile Thiols to the Aromas of White Wines Made From Several *Vitis Vinifera* Grape Varieties. *Am. J. Enol. Vitic.* **2000**, *51*, 178–181. https://doi.org/10.5344/ajev.2000.51.2.178.
